# Micro- and Nanoplastics as Emerging Cardiovascular Risk Factors: A Systematic Review

**DOI:** 10.3390/jox16040131

**Published:** 2026-07-12

**Authors:** Dominika Kaczyńska, Emilia Malik, Kamil Szemik, Szymon Pokrzywiński, Wiktoria Nowojewska, Adam Mitręga, Jakub Kufel

**Affiliations:** 1 Students’ Scientific Association of Computer Analysis and Artificial Intelligence, Department of Radiology and Nuclear Medicine, Medical University of Silesia, 40-055 Katowice, Poland; 2 Department of Biophysics, Faculty of Medical Sciences in Zabrze, Medical University of Silesia, 41-808 Zabrze, Poland; 3 Department of Radiology and Nuclear Medicine, Faculty of Medical Sciences in Katowice, Medical University of Silesia, 40-752 Katowice, Poland

**Keywords:** nanoplastic, microplastic, cardiology, heart diseases, vascular diseases, atherosclerosis

## Abstract

Background: Micro- and nanoplastics (MNPs) are emerging contaminants increasingly detected in human tissues and biological fluids. Their presence in blood, vascular tissues, thrombi, and atherosclerotic plaques raises concern about their possible association with cardiovascular disease. This systematic review synthesized evidence on associations between MNPs and cardiovascular pathology. Methods: A systematic search was conducted in October 2025 in PubMed, Scopus, Web of Science, and Embase according to PRISMA guidelines and a PICOS-based strategy. Original human studies from the last 10 years were eligible. Fourteen studies were included. Due to methodological heterogeneity, a narrative synthesis was performed. Risk of bias was assessed using ROBINS-E, and certainty of evidence was evaluated using a GRADE-informed approach. Results: MNPs were detected in multiple cardiovascular-related matrices. Included studies suggested possible associations with major adverse cardiovascular events, acute coronary syndrome, myocardial infarction, arterial stenosis, vascular calcification, thromboembolic disease, hypertension, inflammatory markers, coagulation-related parameters, and lipid profiles. However, the certainty of evidence was very low, and most studies had a high or very high risk of bias. Conclusions: Current evidence suggests a possible association between MNPs and cardiovascular pathology, but causality remains unproven. Larger prospective studies using standardized detection protocols, rigorous contamination control, and adjustment for confounders are needed.

## 1. Introduction

As plastic production continues to increase, global contamination by micro- and nanoplastics (MNPs) has become a significant public health concern [1,2]. Because these polymers are widespread in the environment, human exposure is chronic and difficult to avoid [3]. Research indicates that synthetic particles enter the body through multiple pathways: primarily via the gastrointestinal tract through contaminated water and food, but also through the respiratory system via inhalation and through direct skin contact [4]. With the current state of the literature, the exact links between human health and microplastic presence in the environment are not fully specified, but the progression of environmental pollution and standardization of detection methods in the near future could bring different conclusions, as there are significant gaps in current knowledge [3,5].

In the toxicological literature, it is crucial to differentiate these structures based on their size. Microplastics (MPs) are commonly defined as particles ranging from 1 µm to 5 mm, while nanoplastics (NPs) represent the fraction below 1 µm [6]. Reducing polymer size to the nanometer scale significantly alters their biological behavior. While larger plastic particles, over 1 µm, exert toxicity mainly through mechanical tissue damage, NPs have been shown to directly penetrate cells via endocytosis and breach physiological barriers, such as the intestinal epithelium or the pulmonary barrier, enabling NPs to enter systemic circulation [1,7].

The consequence of this translocation is the systemic distribution of absorbed polymers [4]. Contemporary tissue analyses have demonstrated the presence of MNPs in numerous human organs and body fluids, including lung parenchyma, feces, breast milk, and the placenta, suggesting a risk of significant organ exposure as early as fetal life [1,8]. Evidence suggests that the smallest particles may cross the blood–brain barrier and accumulate in the nervous system tissues [3].

New experimental and in vivo evidence of MNPs gathering in high-flow circulatory structures has raised concerns regarding potential vascular risks [4]. Direct analytical measurements have documented the presence of these contaminants (primarily polyethylene and polyvinyl chloride) in human blood, myocardial tissue, and thrombi and carotid artery atherosclerotic plaques [1]. Importantly, from a clinical perspective, the presence of plastic deposits within an atherosclerotic plaque is associated with a 4.5-fold higher risk of a composite endpoint including myocardial infarction, stroke, or death [7].

The biological mechanisms underlying potential MNP cardiotoxicity are multifaceted and largely based on the foreign body theory [1]. The presence of polymers in the vessel wall may provoke a chronic immune response through the activation and infiltration of macrophages, which intensifies the cascade of pro-inflammatory interleukin and cytokine secretion [7]. The intracellular presence of NPs may disrupt mitochondrial function and contribute to oxidative stress [1]. Damage and dysfunction of the vascular endothelium, combined with the physical presence of foreign bodies, may disrupt coagulation homeostasis, promote local thrombogenesis, and significantly increase the likelihood of atherosclerotic plaque destabilization [4,7].

Despite these emerging scientific reports, data defining the precise role of polymers in the pathophysiology of vascular diseases remain ambiguous and fragmented [7]. A significant research gap is evident in the literature due to the lack of a broad synthesis of clinical data, making it difficult to verify whether the presence of plastic in tissues contributes to cardiovascular incidents or merely serves as a surrogate marker of generalized environmental pollution and the severity of atherosclerosis [1,7].

Given these uncertainties, the objective of this systematic review is to collect and systematically evaluate available human evidence from the last ten years on the potential associations between MNPs and cardiovascular diseases. Such synthesis may help clarify the current state of evidence, identify methodological limitations, and define priorities for future research.

## 2. Materials and Methods

The protocol for this systematic review has been registered in PROSPERO under the registration number CRD420261422430. The protocol is publicly available.

This research was conducted in accordance with the PRISMA Statement guidelines [9] provided in Supplementary Files S1 and S2. The systematic review was evaluated using the JBI Checklist [10] contained in Supplementary File S3.

### 2.1. Search Strategy and Selection Criteria

The database search was performed on 20 October 2025 in PubMed, Scopus, Web of Science, and Embase. The search strategy was based on the PICOS framework provided in Supplementary File S4, which guided the overall search concept, eligibility criteria, and keyword selection. The search combined terms related to micro- and nanoplastics with terms related to cardiovascular, cardiac, and vascular diseases. The search was restricted to studies published within the last 10 years, defined as the period from 2015 to 2025. This time restriction was applied to focus this review on recent evidence because methods used to detect and characterize micro-, submicron-, and nanoplastic particles have changed considerably in recent years. As these techniques remain method-dependent and technically heterogeneous, older studies may not fully reflect current analytical approaches used for detecting micro- and nanoplastics in human biological samples [6,11]. The exact search strings for PubMed, Scopus, Web of Science, and Embase, together with the database-specific filters and the number of records retrieved before and after filtering, are provided in Supplementary File S5.

All records retrieved from the database searches were imported into Rayyan [12] for duplicate removal and screening. Initially, 1139 records were identified across all databases. After applying database-specific filters, 320 records remained. A total of 73 duplicates were removed. Subsequently, two independent researchers conducted a preliminary review of 247 articles based on titles, abstracts, and keywords. During title and abstract screening, 231 records were excluded because they did not meet the predefined eligibility criteria. As individual records could meet more than one exclusion criterion, detailed exclusion categories are presented in the PRISMA flow diagram in Supplementary File S6. Conflicts were resolved by a third independent researcher. After title and abstract screening, 16 full-text reports were assessed for eligibility. Of these, 14 studies met the inclusion criteria and were included in the systematic review. Two full-text reports were excluded due to wrong study designs. The complete study selection process, including reasons for exclusion at the full-text stage, is presented in the PRISMA flow diagram in Supplementary File S6. Inter-rater reliability for the screening process was assessed using Cohen’s kappa test. The kappa value was 0.641, indicating substantial agreement [13].

### 2.2. Inclusion and Exclusion Criteria

Original human studies investigating the potential association between microplastics or nanoplastics and cardiovascular diseases were eligible for inclusion. Cardiovascular outcomes and conditions included cardiac and vascular pathologies, such as atherosclerosis, thrombotic disease, myocardial infarction, acute coronary syndrome, vascular stenosis, vascular calcification, hypertension, and major adverse cardiovascular events.

Both prospective and retrospective observational studies were considered eligible. Studies were included if they reported original human data, addressed micro- or nanoplastic detection or exposure, and were relevant to cardiovascular, cardiac, or vascular outcomes. Eligible studies were required to have an abstract and an available full text.

The exclusion criteria included pediatric populations, non-human or animal-only studies, in vitro-only studies without original human data, non-original publications, systematic reviews, narrative reviews, meta-analyses, editorials, letters, conference abstracts, and studies not addressing cardiovascular outcomes. Publications older than 10 years were excluded.

### 2.3. Data Extraction

The following data were extracted by three independent analysts from the selected articles. Based on the common criteria identified during phase 1 of data extraction, the following items were collected: 1. general characteristics: authors, publication date, country, journal, impact factor, number of citations, source of funding, and keywords; 2. study design and population: type of study, sample size, sex distribution, mean/median age, and anatomical location of sampling; 3. methodology: relevance of microplastics to the study objective, experimental procedure or exposure assessment, and method of microplastic detection; 4. microplastic characteristics: polymer types detected, particle size, and additional findings; and 5. outcomes: main study results, conclusions, and study limitations. Due to the methodological heterogeneity of the included studies, a meta-analysis was not performed, and a narrative synthesis of the results was conducted instead. In addition, for each analytical study, we extracted information on the statistical model used, confounders included in the analysis, important unmeasured confounders, and potential reverse causation concerns. These data are presented in Supplementary File S7.

### 2.4. Risk-of-Bias Assessment

The methodological quality and risk of bias of the included studies were assessed independently by two reviewers using the ROBINS-E tool [14], which is designed for non-randomized studies of exposure effects. Disagreements were resolved through consensus or consultation with a third reviewer. The assessed domains included bias due to confounding, exposure measurement, participant selection, post-exposure interventions, missing data, outcome measurement, and selection of the reported result. The results are presented as traffic light plots generated using the robvis tool [15].

### 2.5. Certainty-of-Evidence Assessment

We performed a GRADE-informed certainty-of-evidence assessment across the main outcome domains, following the general principles of the GRADE approach [16,17]. Because of substantial heterogeneity in study designs, biological matrices, analytical methods, and reported outcomes, a formal meta-analysis was not performed. Therefore, certainty was assessed narratively at the outcome domain level rather than through a formal meta-analytic GRADE approach. Observational evidence was initially considered low-certainty. The certainty of evidence was then assessed using the main GRADE domains: risk of bias, inconsistency, indirectness, imprecision, and publication bias [16,17]. Evidence was downgraded when concerns were identified in these domains. The final certainty of evidence was classified as high, moderate, low, or very low. The detailed GRADE-informed certainty assessment is provided in Supplementary File S8.

## 3. Results

### 3.1. Risk-of-Bias Assessment

**Figure 1:**
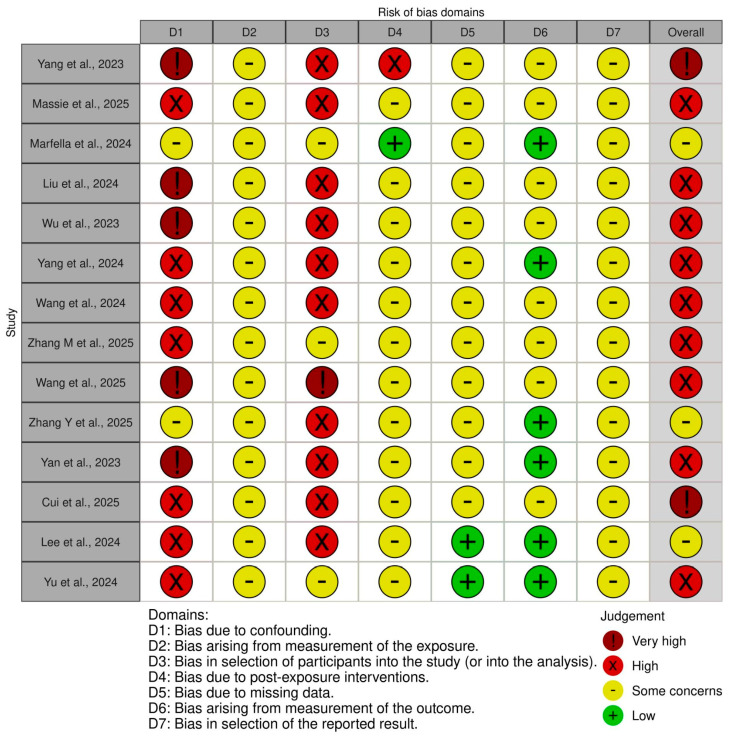
Traffic light plot for the included studies [7,18,19,20,21,22,23,24,25,26,27,28,29,30] assessed using the ROBINS-E tool [14,15].

The risk-of-bias assessment showed that the majority of the included studies had a high or very high risk of bias, with none judged to be at low risk. The lowest risk was observed in prospective observational studies with clinical follow-up and predefined cardiovascular outcomes, particularly by Marfella et al. and Zhang et al. [7,18]. However, these studies still raised some concerns due to residual confounding, selected clinical populations, and the inability to establish causality. Most remaining studies were cross-sectional, case–control, or descriptive detection designs, and were mainly limited by small sample sizes, lack of temporality between microplastic exposure and cardiovascular outcomes, limited adjustment for confounders, and potential contamination during sample collection or laboratory processing [7,18,19,20,21,22,23,24,25,26,27,28,29,30]. Additional concerns stemmed from the methodological limitations of microplastic detection techniques, including limited data on particle size, morphology, or nanoplastic burden [7,18,19,20,21,22,23,24,25,26,27,28,29,30]. Overall, the current findings should be interpreted as preliminary and hypothesis-generating rather than conclusive evidence of a causal relationship between microplastic exposure and cardiovascular disease. A visualization of the domain-level risk-of-bias judgements is presented in Figure 1.

### 3.2. Confounder Adjustment and Reverse Causation

Information on the statistical models, adjusted confounders, important unmeasured confounders, and reverse causation concerns is summarized in Supplementary File S7. Confounder adjustment varied substantially across studies. Some studies used multivariable models adjusted for selected demographic or clinical variables, whereas others relied mainly on descriptive analyses, group comparisons, or correlation analyses. Important factors such as diet, socioeconomic status, occupational exposure, medication use, comorbidities, and iatrogenic or procedure-related exposure were often not fully addressed.

### 3.3. Certainty of Evidence

The GRADE-informed certainty of evidence assessment is summarized in Supplementary File S8. Overall, the certainty of evidence was very low across the main outcome domains. This was mainly due to small sample sizes, observational and cross-sectional study designs, heterogeneous biological matrices and outcomes, inconsistent adjustment for confounding, and concerns regarding reverse causation. Therefore, the available evidence supports possible associations between MNP or MP detection and cardiovascular or related outcomes, but does not establish causality.

### 3.4. Characteristics of Included Studies

To better contextualize the existing clinical evidence, we summarized the methodological characteristics of all included studies in Table 1.

### 3.5. Detection of Micro- and Nanoplastics in Cardiovascular Tissues

The clinical associations between micro- and nanoplastic burden and cardiovascular outcomes are summarized in Supplementary File S7.

### 3.6. Localization of Microplastics in Human Tissues

In the circulatory system, polyethylene (PE) is frequently identified as a dominant polymer, detected in coronary blood and venous samples from both cardiac patients and healthy volunteers [23,27,28]. Studies on extracranial artery stenosis and myocardial infarction have also highlighted polyvinyl chloride (PVC) and polyamide 66 (PA66) as prevalent mass concentration components in the blood [18,29]. Furthermore, polystyrene (PS) and polypropylene (PP) have been quantified in the blood of healthy adults [28], while polyethylene terephthalate (PET) and polyamide (PA) were detected in preoperative samples from cardiac surgery patients [19]. Atherosclerotic plaques appear to act as long-term repositories for micro- and nanoplastics (MNPs), often exhibiting higher concentrations than circulating blood. PE remains a primary contaminant in carotid and femoral plaques [7,20]. However, tissue-specific profiles also show significant levels of PP in carotid plaques [27] and PET in arterial tissues [21]. Analysis of surgically retrieved thrombi from cerebral, coronary, and venous systems identified PE and PA66 as major components [24]. In fecal matter, PS accounted for over 42% of particles in patients with vascular calcification [26]. In hypertensive cohorts, PA, polyurethane (PU), and chlorinated polyethylene (CPE) were the most prevalent polymers in fecal samples [25]. Additionally, PE, PVC, and polycarbonate (PC) have been detected in the umbilical cord blood [30].

### 3.7. Association Between Micro- and Nanoplastics and Clinical Cardiovascular Outcomes

#### 3.7.1. Association with Major Adverse Cardiovascular Events (MACEs)

Marfella et al. demonstrated that patients with MNP-positive atherosclerotic plaques had a 4.53-fold higher incidence of myocardial infarction (MI), ischemic stroke (IS), or death [7]. Yang et al. found that blood MNP levels were higher in patients with acute coronary syndrome (ACS) than in controls and increased with disease severity, with the highest concentrations observed in patients with MI compared with those with unstable angina (UA) [23]. Zhang et al. showed that higher PVC levels in patients with MI were associated with an increased probability of major adverse cardiovascular events (MACEs). Specifically, patients who experienced MACE exhibited higher PVC levels, and elevated PVC levels in thrombi were also associated with a greater likelihood of MACEs after MI. Moreover, each 10-unit increase in PVC was associated with a 1.374-fold increase in MACE odds [18].

#### 3.7.2. Association with Plaques and Arterial Calcification

Marfella et al. detected PE in carotid artery plaque in 150 patients (58.4%), with a mean level of 21.7 ± 24.5 μg/mg, and PVC in 31 patients (12.1%) with a mean level of 5.2 ± 2.4 μg/mg. Additionally, scanning electron microscopy (SEM) revealed visible, jagged-edged MNPs among plaque macrophages and in the extracellular space as scattered debris [7]. Massie et al. reported an approximately 80-fold higher MNP concentration in femoral plaques than in control carotid tissue (3234 ± 3629 μg/g vs. 40.68 ± 23.23 μg/g) [20], while Liu et al. detected MNPs in all arterial samples, with higher levels in coronary and carotid arteries than in plaque-free aorta (coronary arteries: 156.50 μg/g; carotid arteries: 133.37 μg/g vs. aorta: 76.26 μg/g) [21]. Cui et al. found tissue-specific microplastic (MP) accumulation profiles in plaques, with a median concentration of 432.9 µg/g (131.4–1718.8) in carotid atherosclerotic plaque and 75.2 μg/g (12.1–303.2) in blood [27]. Yang et al. revealed that total MNPs positively correlated with greater coronary lesion complexity assessed by the SYNTAX score [23], and Yu et al. showed that MNP concentrations were associated with more severe arterial stenosis (*p* < 0.001). Additionally, they reported that MNP concentrations were significantly higher in patients with extracranial carotid artery stenosis (ECAS) than in controls (174.89 ± 24.95 µg/g vs. 79.82 ± 31.73 µg/g) [29]. In a study by Yan et al., patients with vascular calcification (VC) had higher fecal levels of total MPs, polypropylene (PP), and polystyrene (PS) than patients without VC [26].

#### 3.7.3. Association with Hypertension

Zhang et al. detected microplastics in all 45 umbilical cord samples, and the total concentration was significantly higher in the pregnancy-induced hypertension (PIH) group [30]. Wang et al. observed that individuals with hypertension had higher fecal microplastic concentrations than controls [25].

#### 3.7.4. Other Findings

Yang et al. detected MPs in cardiac and pericardial tissues in patients undergoing cardiac surgery. They also detected MNPs in paired preoperative and postoperative blood samples and observed changes in the type and size distribution of MNPs after the intervention [19].

### 3.8. Mechanistic and Pathophysiological Evidence

#### 3.8.1. Inflammatory Activation

Marfella et al. demonstrated that the presence of MNPs, especially PE, in atherosclerotic plaques was associated with higher levels of local inflammatory markers, including IL-18, IL-1β, IL-6, TNF-α, and expression of CD3 and CD68 [7]. Yang et al. showed that a higher total MNP burden in the blood of patients with ACS was associated with elevated levels of IL-6, IL-12p70, B lymphocytes, and NK cells. Heterogeneous associations were observed in the analysis of individual MNP types [23]. In the study by Zhang et al., higher PVC concentrations in the coronary blood of patients with MI were associated with a higher likelihood of MACEs and with higher levels of IL-1β, IL-6, IL-18, and TNF-α. In addition, PVC in coronary thrombi was positively correlated with inflammatory markers and with monocyte–macrophage infiltration in blood [18]. In the study by Lee et al., a higher total number of MNPs in the blood was positively associated with higher hsCRP levels [28].

#### 3.8.2. Coagulation, Thromboembolic, and Lipid Biomarkers

Wu et al. and Wang et al. demonstrated the presence of MNPs in thrombus and together with other studies evaluated the association with coagulation and thromboembolic markers [22,24]. Wang et al. reported that MPs detected in thrombi were associated with higher D-dimer levels [24]. Similarly, in the study by Yu et al., the ECAS group, associated with higher MNPs, showed higher D-dimer levels and a prolonged thrombin time [29]. Moreover, higher MNP levels are associated with prolonged aPTT, a higher fibrinogen concentration, and higher hsCRP in blood [29]. In addition, MP levels in thrombi were positively associated with platelet count [22]. Cui et al. reported associations between higher concentrations of MPs in blood and adverse lipid profiles, including elevated triglycerides, LDL-C, and ApoB, alongside reduced HDL-C [27]. However, the studies by Wang et al., Wu et al. and Yang et al. failed to demonstrate any significant correlation between MNP levels and changes in lipid profile [22,23,24].

### 3.9. Heterogeneity in Methodology, Detection Methods and Quantification

Substantial methodological heterogeneity was observed across the included studies. The analyzed biological matrices varied widely, including atherosclerotic plaques, arterial tissue, venous or coronary blood, thrombi, fecal samples, umbilical cord blood, and cardiac tissue provided in the Supplementary File S7. The detection methods were likewise inconsistent, comprising Py-GC/MS, LDIR, FTIR, Raman spectroscopy, SEM or combinations thereof [Table 1]. Owing to discrepancies in the detection methods, studies reported different endpoints, including polymer mass, particle counts, and morphological characteristics. As a result, absolute concentrations could not be directly compared across studies. Therefore, the measurement results should be interpreted as method-dependent and not as directly comparable values.

Pyrolysis–gas chromatography/mass spectrometry (Py-GC/MS) is the gold standard for quantifying mass concentrations and identifying polymer types [20]. However, samples are destroyed during the process, which renders further sample analysis impossible. Furthermore, lipid interference can lead to biased results for polyethylene (PE) and polyvinyl chloride (PVC) [20,27,29].

To assess physical properties, laser direct infrared (LDIR) spectroscopy provides rapid data on particle count and morphology, although it is limited by a 20 μm detection threshold and protein interference, especially with PA 66 [19,24,27,29]. For sub-micron analysis, Raman spectroscopy offers superior resolution, down to 360 nm, and pigment identification, but is hindered by slow processing speeds, fluorescence, and surface biofouling [22,24].

Morphological details and visual data at the nanoscale, down to 1 nm, are obtained via scanning electron microscopy (SEM), although this technique requires complex preparation and lacks standalone chemical identification [29]. Finally, stable carbon isotope analysis serves as a complementary tool to definitively differentiate petrochemical-derived plastics from biological tissues [7].

FTIR (Fourier-transform infrared spectroscopy) and µFTIR (micro-Fourier-transform infrared spectroscopy) are both methods based on infrared photon absorption [24,26,28]. µFTIR includes the use of optical microscopes [28]. Both techniques allow for the identification of organic materials by comparing them with the infrared absorption spectra of known substances from a spectral library [28]. µFTIR also allows for analysis of the surfaces of materials, which could be used to analyze plastic decomposition [29].

A comparison of the detection methods used in the studies is presented in Table 2.

#### Sources of MNP Exposure and Sample Contamination

In the study by Yang et al., surgical equipment was identified as the primary source of MNPs, with measurements conducted before and after surgery [19]. Other studies used questionnaires to assess associations between elevated microplastic levels and lifestyle factors [30]. Zhang et al. reported an association between increased MNP levels in the umbilical cord of pregnant women with PIH and the use of plastic bottles, plastic cutlery, and plastic-packaged takeout meals [30]. Yan et al. reported similar findings, with dust exposure identified as an additional associated factor [26]. Lee et al. found a specific association between elevated blood MNP levels and storing food in plastic containers in the refrigerator [28].

Yu et al. and Wang et al. found no significant correlation between questionnaire-assessed plastic exposure and elevated MNP levels [24,29].

Sample contamination represents a critical challenge in the accurate detection and quantification of micro- and nanoplastics (MNPs). To ensure data integrity, researchers employ various contamination-prevention protocols, primarily based on five core strategies:

- Procedural blanks: These quality-control samples are essential for monitoring, identifying, and accounting for background contamination derived from the environment, equipment, or laboratory procedures, thereby establishing a clean baseline. In practice, these blanks mimic the environmental exposure of the actual samples, for example, by being opened concurrently during tissue retrieval, and undergo identical processing steps, including the same exposure times and reagent volumes.
- Non-plastic materials: Protocols generally require the use of non-plastic consumables and tools, with preference given to steel, titanium, glass, or borosilicate glass containers.
- Environmental control: Procedural steps are ideally restricted to enclosed, controlled, plastic-free, and dust-free environments, such as laminar flow hoods or dedicated cleanrooms. Sample storage conditions should also minimize the risk of contamination.
- Reagent and vessel preparation: All reagents are pre-filtered, and collection vessels are rinsed with ultrapure water or ethanol.
- Validated polymer identification protocols: Reproducible procedures are used to ensure correct polymer identification.

Although the use of procedural blanks was widely adopted across the analyzed studies, the stringency of these controls varied considerably. For instance, some studies supplemented standard procedures with specific tissue blanks, such as titanium rods and ultrapure water designed to simulate blood sample matrices [19,22].

Furthermore, reporting standards regarding plastic-free protocols remain inconsistent. While most authors declared adherence to a plastic-free workflow, minor details concerning ubiquitous laboratory consumables, such as the potential use of single-use plastic pipettes, were frequently omitted. This lack of detail raises questions regarding the restrictiveness of the implemented protocols. Similarly, environmental controls showed substantial inter-study variation, with several authors failing to report specific information on laboratory preparation or air filtration measures [20,24,30]. This further underscores the need for transparent reporting and standardized measures to minimize procedural contamination, which remains nearly unavoidable in microplastic research.

Most studies applied a multimodal approach to validation. Authors used spectral libraries to ensure correct polymer identification, with spectral matching thresholds ranging from 0.65 to 0.80 for LDIR and a hit quality index >70 for Raman and FTIR spectroscopy [22,23,26,29]. Py-GC/MS was validated using calibration curves, with defined linearity and detection limits [19,20,21,29]. To validate the efficiency of sample preparation, including digestion and filtration, researchers performed spiking recovery experiments, with reported recovery rates ranging from 80.8% to 107.8% [23]. These validation strategies differed substantially among studies. While this variation is partly explained by heterogeneity in the underlying detection methodologies, it highlights the need for standardized diagnostic and analytical criteria across MNP studies.

## 4. Discussion

Exposure to MNPs is a growing environmental and public health concern, and their presence has been reported in several human tissues and biological fluids [31]. Recent studies have suggested possible links between MNP accumulation and systemic conditions, including stroke, immune dysfunction, reproductive health impairment, and carcinogenicity [1,32,33]. This review highlights potential associations between MNP exposure and the development or progression of selected cardiovascular conditions. Specifically, current data indicate that MNPs are detectable in multiple cardiovascular-related matrices and may be associated with atherosclerosis, thrombosis, CVD, elevated occurrence of MACE and other cardiovascular conditions.

However, the interpretation of these findings is strongly limited by the quality of the available evidence. Most included studies had a high or very high risk of bias, and none were judged to be at low risk. Therefore, the observed associations should be considered preliminary and hypothesis-generating rather than confirmatory. In particular, residual confounding and reverse causation remain major concerns. Factors such as age, sex, lifestyle, comorbidities, medication use, occupational or environmental exposure, hospitalization, and previous medical procedures may influence both cardiovascular risk and MNP burden. Moreover, the current evidence does not clarify whether MNPs contribute to cardiovascular disease, accumulate secondarily in already damaged or inflamed tissues, or primarily act as markers of cumulative exposure and disease severity.

MNPs have been detected in diverse tissue samples, such as atherosclerotic plaques, arterial walls, blood, thrombi and cardiac and pericardial tissues, with data suggesting a preference for accumulation in diseased tissues [7,18,19,20,21,22,23,24,27,28,29]. Experimental data indicate that MNPs internalized by vascular or endothelial cells disrupt redox homeostasis, increase ROS production, and impair mitochondrial function, thereby activating inflammatory and proapoptotic pathways that contribute to vascular remodeling and fibrosis [34,35,36,37]. Similar mechanisms have also been observed in cardiac models, where polystyrene microplastics and nanoplastics induced myocardial fibrosis, cardiomyocyte apoptosis, and elevated ROS production [38,39,40]. Together, these findings suggest that MNPs may preferentially accumulate in already damaged tissues and potentially amplify ongoing pathological processes. This issue is particularly relevant for atherosclerotic plaques, thrombi, and diseased vascular walls, where MNPs may be retained or detected more easily as a consequence of pre-existing tissue injury or inflammation.

The potential clinical relevance of MNP accumulation is suggested by studies reporting associations between MNP presence and adverse cardiovascular outcomes. Patients with MNP-positive plaques had a higher incidence of myocardial infarction, ischemic stroke, or death, while higher MP burdens were reported in patients with ACS and MI [7,23]. Higher PVC burden was also associated with increased odds of MACEs in patients with MI [18]. These findings may indicate that MNPs are related to vascular disease severity or plaque vulnerability. However, because the evidence is observational, it remains unclear whether MNPs are independent contributors to cardiovascular events, markers of advanced disease, or indicators of cumulative environmental or medical exposure. Preliminary clinical and translational findings also link MNPs to hypertension and PIH, but these associations require confirmation in larger, prospectively designed studies [25,30].

In addition to its potential relationship with vascular morphology and atherosclerotic changes, MNPs may also be linked to thrombotic processes. MNPs were detected in thrombi in patients with IS, MI and DVT, where they correlated with greater disease severity [24]. Moreover, a higher MNP burden has been associated with elevated levels of coagulation- and thrombosis-related markers, including D-dimer, fibrinogen, platelet counts, and prolonged aPTT [22,24,28,29]. These findings are compatible with a possible association between MNP burden and a prothrombotic profile, but they do not establish whether MNPs directly promote thrombosis or are more frequently detected in patients with more severe vascular disease, a hypothesis further supported by in vitro and animal-model studies [34,35,41].

In several studies, MNP accumulation was also associated with immune activation. MNP-positive plaques showed elevated levels of interleukins, TNF-α, CD3 and CD68 cells, B lymphocytes and NK cells [7,23]. In MI patients, PVC levels in coronary blood and thrombi were associated with inflammatory cytokines and monocyte–macrophage infiltration and higher CRP [18,30]. These findings support a possible role of MNPs in promoting an inflammatory vascular environment. Given the established role of inflammation in atherogenesis and plaque progression, MNP-related inflammatory activation may represent a biologically plausible pathway linking MNP exposure to vascular remodeling and atherosclerotic disease [42].

Several MNP polymers were associated with an adverse lipid profile characterized by elevated triglycerides, LDL-C, and ApoB, alongside decreased HDL-C [27]. Such lipid abnormalities may create an environment conducive to atherosclerosis by increasing the availability of atherogenic lipoproteins, which can be taken up by macrophages and promote foam cell formation [43]. Experimental non-human studies further indicate that polystyrene NPs may disrupt lipid metabolism, promote macrophage lipid accumulation and foam-cell formation, and accelerate atherosclerotic lesion development [44,45].

Potential sources of MNP exposure should also be considered when interpreting cardiovascular findings. Some of the studies included in this review attempted to find a correlation between lifestyle and the amount of plastic detected; however, the available evidence remains insufficient to draw definitive conclusions [24,26,28,30]. Questionnaire human fecal studies suggest that consumption of plastic-packaged drinking water or food and exposure to dust may contribute to MNP exposure [46]. This is supported by direct analyses of bottled water and take-out food containers, which indicate that plastic packaging may contain or release substantial numbers of MNPs [47,48]. Medical procedures may also represent an underestimated, iatrogenic source of MNP exposure [19]. In vitro cardiopulmonary bypass studies support this concern, showing that extracorporeal circuits may generate detectable MNPs, although their clinical relevance remains unclear [49].

Large population-based studies can provide a useful epidemiological context for these exposure pathways. For example, Dolcini et al. analyzed nationwide cross-sectional data on bottled plastic water consumption and chronic diseases, including hypertension, using data from 45,597 participants. Such findings could support the public-health relevance of plastic-related exposure assessment at the population level. However, because this evidence is cross-sectional and based on exposure proxies rather than direct MNP quantification in cardiovascular tissues, it should be interpreted cautiously and cannot establish a direct causal pathway from bottled water consumption to cardiovascular events [50].

It is critical to highlight that the substantial variations in detection methods and anti-contamination measures across the current literature hinder accurate comparisons between studies. Recent analytical reviews suggest that combining different techniques yields the most reliable results, with mass-based assessment offering higher verifiability than simple particle counting. Because micro- and nanoplastics can contaminate a sample at any point during preparation or analysis, rigorous quality control protocols are absolutely essential to guarantee honest, reproducible data that accurately reflects internal patient exposure rather than background noise [1].

The growing body of data suggests harmful effects of MNPs on the human body. Cornelli et al. recently proposed defining an emerging ‘Microplastic Syndrome.’ Driven by systemic MNP accumulation, this clinical entity encompasses various multi-organ symptoms and is validated via a structured clinical questionnaire [51]. Despite that, significant knowledge gaps still hinder comprehensive risk assessment. The main limitations of the current data are the limited number of large, well-characterized human studies, as well as the lack of standardized definitions and analytical methods for MNP detection, which significantly limit the comparability of study results. To address these gaps, future research should adopt a multidisciplinary approach and well-designed prospective human studies assessing the associations of MNPs with the course and prognosis of CVD. Prospective patient cohorts with precise assessment of MNPs exposure, standardized detection methods, and rigorous consideration of important confounding factors such as age, lifestyle, comorbidities, pharmacotherapy, and iatrogenic exposure are particularly needed.

### Limitations

The findings of this review should be interpreted with caution due to several recurring methodological limitations across the included studies, which were reflected in the overall risk-of-bias assessment. A major concern was the small and often highly selected study populations in 10 of the 14 included studies [19,20,21,22,24,25,26,27,28,29]. This reduced statistical power, limited subgroup analyses, and restricted generalizability. Massie et al. reported a marked male predominance, which further limited the interpretation of sex-related differences [20]. Meanwhile, Wang et al. (2025) relied on very limited human data, with most mechanistic conclusions derived from experimental models [25].

Another important limitation was the predominantly observational or cross-sectional design of the evidence base. Half of the articles demonstrated associations between MNP presence and cardiovascular outcomes but could not establish causality or determine whether MNP accumulation preceded disease development [7,22,23,24,25,26,27]. As summarized in Supplementary File S7, residual confounding and reverse causation were recurrent concerns across the included studies, particularly when MNPs or MPs were measured after disease onset or in plaques, thrombi, or diseased vascular tissues. The comparability of results was further limited by substantial methodological heterogeneity, including differences in biological matrices and detection methods. In the studies by Yang et al., Wang et al. and Yu et al., LDIR could not detect particles below 20 μm [19,24,29], and the study by Lee et al. reported similar limitations for µ-FTIR [24]. In the studies by Liu et al. and Cui et al., Py-GC/MS did not provide information on particle size, shape, or morphology [21,27,29]. These limitations were also reflected in the GRADE-informed certainty of evidence assessment, in which the certainty of evidence was rated as very low across the main outcome domains.

Finally, incomplete exposure assessment and possible contamination may have influenced the results. Seven of 14 articles lacked detailed information on dietary, environmental, occupational, or medical sources of exposure [7,18,24,25,26,28,30], while contamination could not be fully excluded in the articles by Yang et al., Marfella et al., Zhang Y et al. and Lee et al. [7,18,23,28].

## 5. Conclusions

This systematic review indicates that MNPs are detectable in several cardiovascular-related tissues and biological matrices, including atherosclerotic plaques, thrombi, blood, and cardiac or pericardial tissues. Across the included studies, MNPs were also reported in circulating blood, fecal samples, and umbilical cord blood, suggesting that their distribution may reflect systemic exposure, tissue-specific accumulation, or retention within already damaged tissues. However, substantial variability in polymer profiles, sampled matrices, analytical methods, and reported concentration measures limits direct comparison between studies.

The reviewed evidence suggests possible associations between MNP presence or burden and cardiovascular outcomes or related biomarkers, including major adverse cardiovascular events, acute coronary syndrome, myocardial infarction, arterial stenosis, vascular calcification, thromboembolic disease, hypertension, inflammatory markers, coagulation-related parameters, and adverse lipid profiles. Experimental and translational data provide biologically plausible mechanisms, including oxidative stress, endothelial dysfunction, inflammatory activation, and altered coagulation. Nevertheless, these findings should be interpreted as preliminary and hypothesis-generating rather than confirmatory.

The strength of these conclusions is limited by the overall quality of the available evidence. In the GRADE-informed assessment, the certainty of evidence was rated as very low across the main outcome domains. This was consistent with the risk-of-bias assessment, in which most included studies had a high or very high risk of bias, and no study was judged to be at low risk. The main limitations included small sample sizes, selected clinical populations, predominantly observational or cross-sectional designs, heterogeneous detection methods, incomplete exposure assessment, limited adjustment for confounders, possible sample contamination, and concerns regarding reverse causation.

At present, it remains unresolved whether MNPs directly contribute to cardiovascular disease, accumulate secondarily in already damaged or inflamed tissues, or primarily serve as markers of cumulative environmental, lifestyle-related, occupational, or medical exposure. Therefore, MNPs should not currently be regarded as established cardiovascular risk factors. Further large-scale prospective human studies are needed, using standardized sampling and analytical protocols, rigorous contamination control procedures, and comprehensive adjustment for key confounding factors, including age, sex, lifestyle, comorbidities, pharmacotherapy, occupational and environmental exposure, and potential iatrogenic sources.

## Figures and Tables

**Table 1:** Characteristics of included studies.

Author	Study Design	Study Population	Participants’ Age	Detection Method
Yang et al., 2023 [19]	Prospective observational study	15 cardiac surgery patients	59.6 years (age span: 41–75)	LDIR; SEM
Massie et al., 2025 [20]	Comparative study	8 patients with common femoral artery plaques; 30 decedent donors (control group)	Femoral artery plaques group mean: 73.8 ± 5.9	Py-GC/MS; F-Search MP software analysis
Marfella et al., 2024 [7]	Prospective, multicenter, observational study	304 patients initially enrolled; 257 on follow up	Group with MNPs: median age was 71 years (IQR: 65–75) Group without MNPs: median age: 73 years (IQR: 67–77)	Py-GC/MS, TEM, SEM, and stable isotope analysis
Liu et al., 2024 [21]	Prospective study	17 patients: 4 with CAD; 7 with carotid atherosclerosis; 6 with aortic dissection	Mean: 63.1 Age range: 49 to 76	Py-GC/MS
Wu et al., 2023 [22]	Observational analytical study	26 patients in total: 24 with arterial dissection; 2 with acute arterial embolism	Mean: 56.5 Age range: 32 to 74	Raman spectrometry, spectral verification, and visual analysis
Yang et al., 2024 [23]	Cross-sectional study	101 patients in total Study group: 82 with ACS Control group: 19 healthy participants	Total population median: 56.85 ± 10.77 ACS group mean: 57.61 Control group mean: 53.58	Py-GC/MS; flow cytometry
Wang et al., 2024 [24]	Multimodal analytical study	30 patients: 16 with ischemic stroke; 5 with MI; 9 with DVT	Mean: 65.2 years	Py-GC/MS, LDIR, and SEM
Zhang et al., 2025 [30]	Preliminary prospective study	45 mother–infant pairs Study group: 15 with PIH Control group: 30 healthy pregnant women	PIH group median: 34.2 ± 3.8 (27–41) Control group median: 32.2 ± 4.3 (21–42)	PY-GC/MS; SEM
Wang et al., 2025 [25]	Human cross-sectional study with an experimental in vivo mouse model (only human research data included)	8 patients in total Study group: 4 with hypertension Control group: 4 healthy participants	Study group median: 53.75 ± 3.09 Control group median: 53.25 ± 1.97	LDIR
Zhang et al., 2025 [18]	Prospective observational study	214 patients with STEMI 110 completed follow-up 21 additional patients with MI for validation	MACE group median: 53.9 ± 7.6 Non-MACE group median: 56.9 ± 6.6	Py-GC/MS, ELISA, and immunohistochemistry
Yan et al., 2023 [26]	Observational clinical study combined with an in vivo experimental rat model (only human research included)	47 patients Study group: 25 with vascular calcification Control group: 22 healthy participants	Total population mean: 39.7 Study group mean: 41.3 Control group mean: 38.0	FTIR; LC-MS
Cui et al., 2025 [27]	Cross-sectional study	20 patients: 12 males; 8 females	Mean age: 66 ± 7	Py-GC/MS
Lee et al., 2024 [28]	Cross-sectional study	36 healthy adults: 10 males; 26 females	Median age: 41	µ-FTIR, µ-Raman analysis, and SEM
Yu et al., 2024 [29]	Prospective observational and comparative study	30 patients who underwent DSA Study group: 20 with vascular stenosis Control group: 10 healthy participants	Total population mean age: 68.47 ± 5.90 Study group mean age: 69.55 ± 5.24 Control group mean age: 66.30 ± 6.82	Py-GC/MS, LDIR, and SEM

Abbreviations: ACS: acute coronary syndrome, CAD: coronary artery disease, DSA: digital subtraction angiography, DVT: deep vein thrombosis, ELISA: enzyme-linked immunosorbent assay, LC-MS: liquid chromatography–mass spectrometry, LDIR: laser direct infrared spectroscopy, MACE: major adverse cardiac event, MI: myocardial infarction, MNPs: microplastics and nanoplastics, PIH: pregnancy-induced hypertension, Py-GC/MS: pyrolysis–gas chromatography–mass spectrometry, SEM: scanning electron microscopy, and TEM: transmission electron microscopy.

**Table 2:** Comparison of detection methods used in the included studies.

Method	Detected Characteristics of Substances	Detection Limit	Main Constraints
Py-GC/MS	Mass concentration and polymer type	N/A (mass-based)	Destructive to the sample, no morphological data, and may slightly change readings for PE, PVC, and PVS due to lipid interference
LDIR	Particle size, count, and shape	~20 μm	Proteins can interfere with outcomes
Raman spectrometry	Chemical identification and pigments	>360 nm	Time intensive
SEM	High-resolution morphology and visual data	~1 nm	No standalone chemical identification
δ13 isotope	Synthetic vs. biological origin of carbon-based materials	N/A	Destructive; highly specialized equipment
FTIR	Infrared absorption spectrum-polymer type	>20 um	Water interferes with outcomes; skilled personnel required
µ-FTIR	Infrared absorption spectrum-polymer type, particle surface analysis, and single-particle analysis	5–20 um	Time-intensive preparation; skilled personnel required

Abbreviations: FTIR: Fourier-transform infrared spectroscopy, LDIR: laser direct infrared imaging, PE: polyethylene, Py-GC/MS: pyrolysis–gas chromatography–mass spectrometry, PVC: polyvinyl chloride, PVS: polyvinyl sulfate, SEM: scanning electron microscopy, and µ-FTIR: micro-Fourier-transform infrared spectroscopy.

## Data Availability

No new data were created or analyzed in this study. Data sharing is not applicable to this article.

## Supplementary Materials

The following supporting information can be downloaded at https://www.mdpi.com/article/10.3390/jox16040131/s1. File S1: PRISMA 2020 Checklist; File S2: PRISMA 2020 Abstract Checklist; File S3: JBI Critical Appraisal Checklist; File S4: PICOS framework; File S5: Detailed search strategy; File S6: PRISMA flow diagram; File S7: Types and localization of microplastics in specimens, confounders, and reverse causation concern; File S8: GRADE-informed certainty assessment. References [7,18,19,20,21,22,23,24,25,26,27,28,29,30,52] are cited in the Supplementary Materials.

## Abbreviations

The following abbreviations are used in this manuscript:

ACS	Acute coronary syndrome
ApoB	Apolipoprotein B
APTT	Activated partial thromboplastin time
CD3	Cluster of differentiation 3
CD68	Cluster of differentiation 68
CPE	Chlorinated polyethylene
CRP	C-reactive protein
CVD	Cardiovascular disease
DSA	Digital subtraction angiography
DVT	Deep vein thrombosis
ECAS	Extracranial carotid artery stenosis
ELISA	Enzyme-linked immunosorbent assay
FTIR	Fourier-transform infrared spectroscopy
HDL-C	High-density lipoprotein cholesterol
hsCRP	High-sensitivity C-reactive protein
IL-1β	Interleukin-1 beta
IL-6	Interleukin-6
IL-12p70	Interleukin-12 p70
IL-18	Interleukin-18
IQR	Interquartile range
IS	Ischemic stroke
JBI	Joanna Briggs Institute
LC-MS	Liquid chromatography–mass spectrometry
LDIR	Laser direct infrared spectroscopy
LDL-C	Low-density lipoprotein cholesterol
MACE	Major adverse cardiovascular event
MI	Myocardial infarction
MNPs	Micro- and nanoplastics
MPs	Microplastics
NK	Natural killer
NPs	Nanoplastics
PA	Polyamide
PA66	Polyamide 66
PC	Polycarbonate
PE	Polyethylene
PET	Polyethylene terephthalate
PICOS	Population, Intervention, Comparison, Outcomes, and Study design
PIH	Pregnancy-induced hypertension
PP	Polypropylene
PRISMA	Preferred Reporting Items for Systematic Reviews and Meta-Analyses
PS	Polystyrene
PU	Polyurethane
PVC	Polyvinyl chloride
Py-GC/MS	Pyrolysis–gas chromatography–mass spectrometry
ROBINS-E	Risk of Bias in Non-Randomized Studies of Exposures
ROS	Reactive oxygen species
SEM	Scanning electron microscopy
STEMI	ST-segment elevation myocardial infarction
SYNTAX	Synergy Between Percutaneous Coronary Intervention with Taxus and Cardiac Surgery
TEM	Transmission electron microscopy
TNF-α	Tumor necrosis factor alpha
UA	Unstable angina
VC	Vascular calcification
µ-FTIR	Micro-Fourier-transform infrared spectroscopy
µ-Raman	Micro-Raman spectroscopy

## References

[1] Zheng H., Vidili G., Casu G., Navarese E.P., Sechi L.A., Chen Y. (2024). Microplastics and Nanoplastics in Cardiovascular Disease—A Narrative Review with Worrying Links. Front. Toxicol..

[2] World Health Organization (2022). Dietary and Inhalation Exposure to Nano- and Microplastic Particles and Potential Implications for Human Health.

[3] Vethaak A.D., Legler J. (2021). Microplastics and Human Health. Science.

[4] Zhu Y., Che R., Zong X., Wang J., Li J., Zhang C., Wang F. (2024). A Comprehensive Review on the Source, Ingestion Route, Attachment and Toxicity of Microplastics/Nanoplastics in Human Systems. J. Environ. Manag..

[5] (2019). Science Advice for Policy by European Academies. A Scientific Perspective on Microplastics in Nature and Society.

[6] Caldwell J., Taladriz-Blanco P., Lehner R., Lubskyy A., Ortuso R.D., Rothen-Rutishauser B., Petri-Fink A. (2022). The Micro-, Submicron-, and Nanoplastic Hunt: A Review of Detection Methods for Plastic Particles. Chemosphere.

[7] Marfella R., Prattichizzo F., Sardu C., Fulgenzi G., Graciotti L., Spadoni T., D’Onofrio N., Scisciola L., La Grotta R., Frigé C. (2024). Microplastics and Nanoplastics in Atheromas and Cardiovascular Events. N. Engl. J. Med..

[8] Ragusa A., Svelato A., Santacroce C., Catalano P., Notarstefano V., Carnevali O., Papa F., Rongioletti M.C.A., Baiocco F., Draghi S. (2021). Plasticenta: First Evidence of Microplastics in Human Placenta. Environ. Int..

[9] Page M.J., Moher D., Bossuyt P.M., Boutron I., Hoffmann T.C., Mulrow C.D., Shamseer L., Tetzlaff J.M., Akl E.A., Brennan S.E. (2021). PRISMA 2020 Explanation and Elaboration: Updated Guidance and Exemplars for Reporting Systematic Reviews. BMJ.

[10] Hilton M. (2024). JBI Critical Appraisal Checklist for Systematic Reviews and Research Syntheses. J. Can. Health Libr. Assoc..

[11] Ye Y., Yu K., Zhao Y. (2022). The development and application of advanced analytical methods in microplastics contamination detection: A critical review. Sci. Total Environ..

[12] Ouzzani M., Hammady H., Fedorowicz Z., Elmagarmid A. (2016). Rayyan—A Web and Mobile App for Systematic Reviews. Syst. Rev..

[13] McHugh M.L. (2012). Interrater Reliability: The Kappa Statistic. Biochem. Medica.

[14] Higgins J.P.T., Morgan R.L., Rooney A.A., Taylor K.W., Thayer K.A., Silva R.A., Lemeris C., Akl E.A., Bateson T.F., Berkman N.D. (2024). A Tool to Assess Risk of Bias in Non-Randomized Follow-up Studies of Exposure Effects (ROBINS-E). Environ. Int..

[15] McGuinness L.A., Higgins J.P.T. (2021). Risk-of-bias VISualization (Robvis): An R Package and Shiny Web App for Visualizing Risk-of-bias Assessments. Res. Synth. Methods.

[16] Guyatt G.H., Oxman A.D., Vist G.E., Kunz R., Falck-Ytter Y., Alonso-Coello P., Schünemann H.J. (2008). GRADE: An Emerging Consensus on Rating Quality of Evidence and Strength of Recommendations. BMJ.

[17] Neumann I., Schünemann H. The GRADE Book.

[18] Zhang Y., Gao Q., Gao Q., Xu M., Fang N., Mu L., Han X., Yu H., Zhang S., Li Y. (2025). Microplastics and Nanoplastics Increase Major Adverse Cardiac Events in Patients with Myocardial Infarction. J. Hazard. Mater..

[19] Yang Y., Xie E., Du Z., Peng Z., Han Z., Li L., Zhao R., Qin Y., Xue M., Li F. (2023). Detection of Various Microplastics in Patients Undergoing Cardiac Surgery. Environ. Sci. Technol..

[20] Massie P.L., Garcia M.A., Gallego D., Schlosser C., Decker A., Liu R., Mazloumi Bakhshayesh M., Kulkarni D., Justus M.P., Pace C. (2025). Micro- and Nanoplastics Are Elevated in Femoral Atherosclerotic Plaques Compared with Undiseased Arteries. JVS-Vasc. Sci..

[21] Liu S., Wang C., Yang Y., Du Z., Li L., Zhang M., Ni S., Yue Z., Yang K., Wang Y. (2024). Microplastics in Three Types of Human Arteries Detected by Pyrolysis-Gas Chromatography/Mass Spectrometry (Py-GC/MS). J. Hazard. Mater..

[22] Wu D., Feng Y., Wang R., Jiang J., Guan Q., Yang X., Wei H., Xia Y., Luo Y. (2023). Pigment Microparticles and Microplastics Found in Human Thrombi Based on Raman Spectral Evidence. J. Adv. Res..

[23] Yang Y., Zhang F., Jiang Z., Du Z., Liu S., Zhang M., Jin Y., Qin Y., Yang X., Wang C. (2024). Microplastics Are Associated with Elevated Atherosclerotic Risk and Increased Vascular Complexity in Acute Coronary Syndrome Patients. Part. Fibre Toxicol..

[24] Wang T., Yi Z., Liu X., Cai Y., Huang X., Fang J., Shen R., Lu W., Xiao Y., Zhuang W. (2024). Multimodal Detection and Analysis of Microplastics in Human Thrombi from Multiple Anatomically Distinct Sites. eBioMedicine.

[25] Wang S., Yan K., Dong Y., Chen Y., Song J., Chen Y., Liu X., Qi R., Zhou X., Zhong J. (2025). The Influence of Microplastics on Hypertension-Associated Cardiovascular Injury via the Modulation of Gut Microbiota. Environ. Pollut..

[26] Yan J., Pan Y., He J., Pang X., Shao W., Wang C., Wang R., He Y., Zhang M., Ye J. (2023). Toxic Vascular Effects of Polystyrene Microplastic Exposure. Sci. Total Environ..

[27] Cui C., Guo Z., Liu Y., Han N., Song J., Chen Y., Zheng Y., Sheng C., Balmer L., Li H. (2025). Tissue-Specific Distribution of Microplastics in Human Blood and Carotid Plaques: A Paired Sample Analysis. Environ. Int..

[28] Lee D.-W., Jung J., Park S., Lee Y., Kim J., Han C., Kim H.-C., Lee J.H., Hong Y.-C. (2024). Microplastic Particles in Human Blood and Their Association with Coagulation Markers. Sci. Rep..

[29] Yu H., Li H., Cui C., Han Y., Xiao Y., Zhang B., Li G. (2024). Association between Blood Microplastic Levels and Severity of Extracranial Artery Stenosis. J. Hazard. Mater..

[30] Zhang M., Zhang Y., Liu T., An C., Sun Y. (2025). Microplastic Exposure in Daily Life and the Risk of Pregnancy-Induced Hypertension: A Study on the Association between Environmental Pollutants and Maternal-Fetal Health Outcomes. J. Hazard. Mater..

[31] Roslan N.S., Lee Y.Y., Ibrahim Y.S., Tuan Anuar S., Yusof K.M.K.K., Lai L.A., Brentnall T. (2024). Detection of Microplastics in Human Tissues and Organs: A Scoping Review. J. Glob. Health.

[32] Kufel J., Korbaś M., Janiec J., Pankowska Z., Młynek M., Gaweł A., Mitręga A. (2026). Micro- and Nanoplastics as a Potential Risk Factor for Stroke: A Systematic Review. J. Xenobiotics.

[33] Ali N., Katsouli J., Marczylo E.L., Gant T.W., Wright S., Bernardino De La Serna J. (2024). The Potential Impacts of Micro-and-Nano Plastics on Various Organ Systems in Humans. eBioMedicine.

[34] Fu Y., Fan M., Xu L., Wang H., Hu Q., Jin Y. (2022). Amino-Functionalized Polystyrene Nano-Plastics Induce Mitochondria Damage in Human Umbilical Vein Endothelial Cells. Toxics.

[35] Basini G., Grolli S., Bertini S., Bussolati S., Berni M., Berni P., Ramoni R., Scaltriti E., Quintavalla F., Grasselli F. (2023). Nanoplastics Induced Oxidative Stress and VEGF Production in Aortic Endothelial Cells. Environ. Toxicol. Pharmacol..

[36] Liang B., Zhong Y., Huang Y., Lin X., Liu J., Lin L., Hu M., Jiang J., Dai M., Wang B. (2021). Underestimated Health Risks: Polystyrene Micro- and Nanoplastics Jointly Induce Intestinal Barrier Dysfunction by ROS-Mediated Epithelial Cell Apoptosis. Part. Fibre Toxicol..

[37] Persiani E., Cecchettini A., Amato S., Ceccherini E., Gisone I., Sgalippa A., Ippolito C., Castelvetro V., Lomonaco T., Vozzi F. (2025). Virgin and Photo-Degraded Microplastics Induce the Activation of Human Vascular Smooth Muscle Cells. Sci. Rep..

[38] Li Z., Zhu S., Liu Q., Wei J., Jin Y., Wang X., Zhang L. (2020). Polystyrene Microplastics Cause Cardiac Fibrosis by Activating Wnt/β-Catenin Signaling Pathway and Promoting Cardiomyocyte Apoptosis in Rats. Environ. Pollut..

[39] Lin P., Tong X., Xue F., Qianru C., Xinyu T., Zhe L., Zhikun B., Shu L. (2022). Polystyrene Nanoplastics Exacerbate Lipopolysaccharide-Induced Myocardial Fibrosis and Autophagy in Mice via ROS/TGF-Β1/Smad. Toxicology.

[40] Lu T., Yuan X., Sui C., Yang C., Li D., Liu H., Zhang G., Li G., Li S., Zhang J. (2024). Exposure to Polypropylene Microplastics Causes Cardiomyocyte Apoptosis Through Oxidative Stress and Activation of the MAPK-Nrf2 Signaling Pathway. Environ. Toxicol..

[41] Vlacil A.-K., Bänfer S., Jacob R., Trippel N., Kuzu I., Schieffer B., Grote K. (2021). Polystyrene Microplastic Particles Induce Endothelial Activation. PLoS ONE.

[42] Libby P. (2002). Inflammation in Atherosclerosis. Nature.

[43] The Role of Lipids and Lipoproteins in Atherosclerosis. Endotext. NCBI Bookshelf. https://www.ncbi.nlm.nih.gov/books/NBK343489/.

[44] Florance I., Chandrasekaran N., Gopinath P.M., Mukherjee A. (2022). Exposure to Polystyrene Nanoplastics Impairs Lipid Metabolism in Human and Murine Macrophages in Vitro. Ecotoxicol. Environ. Saf..

[45] Wen J., Sun H., Yang B., Song E., Song Y. (2024). Long-Term Polystyrene Nanoplastic Exposure Disrupt Hepatic Lipid Metabolism and Cause Atherosclerosis in ApoE^−^/^−^ Mice. J. Hazard. Mater..

[46] Yan Z., Liu Y., Zhang T., Zhang F., Ren H., Zhang Y. (2022). Analysis of Microplastics in Human Feces Reveals a Correlation between Fecal Microplastics and Inflammatory Bowel Disease Status. Environ. Sci. Technol..

[47] Du F., Cai H., Zhang Q., Chen Q., Shi H. (2020). Microplastics in Take-out Food Containers. J. Hazard. Mater..

[48] Qian N., Gao X., Lang X., Deng H., Bratu T.M., Chen Q., Stapleton P., Yan B., Min W. (2024). Rapid Single-Particle Chemical Imaging of Nanoplastics by SRS Microscopy. Proc. Natl. Acad. Sci. USA.

[49] Green J.L., Field D.T., Bennett R., Jenner L.C., Chapman E.C., Sadofsky L.R., Rotchell J.M., Loubani M. (2025). Microplastics in Cardiopulmonary Bypass: Quantification and Characterization of Particles across Systems. Interdiscip. Cardiovasc. Thorac. Surg..

[50] Dolcini J., Chiavarini M., Firmani G., Ponzio E., D’Errico M.M., Barbadoro P. (2024). Consumption of Bottled Water and Chronic Diseases: A Nationwide Cross-Sectional Study. Int. J. Environ. Res. Public Health.

[51] Cornelli U., Casella C., Belcaro G., Cesarone M.R., Marucci S., Rondanelli M., Recchia M., Zanoni G. (2025). Definition of Emerging Microplastic Syndrome Based on Clinical and Epidemiological Evidence: A Narrative Review. Microplastics.

[52] Page M.J., McKenzie J.E., Bossuyt P.M., Boutron I., Hoffmann T.C., Mulrow C.D., Shamseer L., Tetzlaff J.M., Akl E.A., Brennan S.E. (2021). The PRISMA 2020 statement: an updated guideline for reporting systematic reviews. BMJ.

