# Supplementary material for: Micro- and Nanoplastics as Emerging Cardiovascular Risk Factors: A Systematic Review
Source: J Xenobiot. 2026 Jul 12;16(4):131. doi: 10.3390/jox16040131 (PMC13398113; doi:10.3390/jox16040131)
Supplement: Supplementary file 1 [file jox-16-00131-s001.zip › Supplementary File S3 - JBI Critical Appraisal Checklist.pdf]

JBI Critical Appraisal Checklist for Systematic Reviews and Research Syntheses

	Yes	No	Unclear	Not applicabl e
1. Is the review question clearly and explicitly stated?	X	☐	☐	☐
2. Were the inclusion criteria appropriate for the review question?	X	☐	☐	☐
3. Was the search strategy appropriate?	X	☐	☐	☐
4. Were the sources and resources used to search for studies adequate?	X	☐	☐	☐
5. Were the criteria for appraising studies appropriate?	X	☐	☐	☐
6. Was critical appraisal conducted by two or more reviewers independently?	X	☐	☐	☐
7. Were there methods to minimize errors in data extraction?	X	☐	☐	☐
8. Were the methods used to combine studies appropriate?	X	☐	☐	☐
9. Was the likelihood of publication bias assessed?	X	☐	☐	☐
10. Were recommendations for policy and/or practice supported by the reported data?	X	☐	☐	☐
11. Were the specific directives for new research appropriate?	X	☐	☐	☐

Overall appraisal: Include X Exclude ☐ Seek further info ☐

© JBI, 2020. All rights reserved. JBI grants use of these tools for research purposes only. All other enquiries should be sent to jbisynthesis@adelaide.edu.au.
