# Supplementary material for: Micro- and Nanoplastics as Emerging Cardiovascular Risk Factors: A Systematic Review
Source: J Xenobiot. 2026 Jul 12;16(4):131. doi: 10.3390/jox16040131 (PMC13398113; doi:10.3390/jox16040131)
Supplement: Supplementary file 1 [file jox-16-00131-s001.zip › Supplementary File S4 - PICOS framework.pdf]

PICO scheme of searching

**PICO elements**	**Keywords**	**Search terms**	**Search strategy**
**P** (**P**atient or/and **P**opulation)	Adult patients with cardiovascular disease.	Cardiovascular Diseases	cardiology OR Cardiovascular Diseases OR Heart Diseases OR Vascular Diseases
**I** (**I**ntervention)	Presence of microplastic in the results.	Microplastic	microplastics OR nanoplastics
**C** (**C**omparison)	not applicable	not applicable	not applicable
**O** (**O**utcome)	not applicable	not applicable	not applicable
