# Supplementary material for: Micro- and Nanoplastics as Emerging Cardiovascular Risk Factors: A Systematic Review
Source: J Xenobiot. 2026 Jul 12;16(4):131. doi: 10.3390/jox16040131 (PMC13398113; doi:10.3390/jox16040131)
Supplement: Supplementary file 1 [file jox-16-00131-s001.zip › Supplementary File S5 - Detailed search strategy.pdf]

Search strategy (20.10.2025)

The database search was performed on 20 October 2025. The following databases were searched: PubMed, Scopus, Web of Science, and Embase. The search strategy combined terms related to micro- and nanoplastics with terms related to cardiovascular, cardiac, and vascular diseases. The date restriction was applied across databases and covered publications from 2015 to 2025.

PubMed

Search string:
(microplastics OR nanoplastics) AND (cardiology OR Cardiovascular Diseases OR Heart Diseases OR Vascular Diseases)

Filters applied:
Abstract available; full text available; publication date from 2015 to 2025; humans.

Records identified before filters: 196
Records identified after filters: 91

Scopus

Search string:
TITLE-ABS-KEY((microplastics OR nanoplastics) AND (cardiology OR Cardiovascular Diseases OR Heart Diseases OR Vascular Diseases))

Filters applied:
Publication years 2015–2025, applied as PUBYEAR > 2014 AND PUBYEAR < 2026; article document type, applied as LIMIT-TO(DOCTYPE, "ar").

Records identified before filters: 165
Records identified after filters: 74

Web of Science

Search string:
(microplastics OR nanoplastics) AND (cardiology OR Cardiovascular Diseases OR Heart Diseases OR Vascular Diseases)

Filters applied:
Publication date from 2015 to 2025; article document type.

Records identified before filters: 118
Records identified after filters: 63

Embase

Search string:
('microplastics'/exp OR microplastics OR 'nanoplastics'/exp OR nanoplastics) AND ('cardiology'/exp OR cardiology OR 'cardiovascular diseases'/exp OR 'cardiovascular diseases' OR (('cardiovascular'/exp OR cardiovascular) AND ('diseases'/exp OR diseases)) OR 'heart diseases'/exp OR 'heart diseases' OR (('heart'/exp OR heart) AND ('diseases'/exp OR diseases)) OR 'vascular diseases'/exp OR 'vascular diseases' OR (vascular AND ('diseases'/exp OR diseases)))

Filters applied:
Publication date from 2015 to 2025; article document type; adults.

Records identified before filters: 660
Records identified after filters: 92

Results
Records identified before filtering: 1139
Records remaining after database-specific filters: 320
Records imported into Rayyan before duplicate removal: 320
Duplicates removed: 73
Records screened by title and abstract: 247
The conflict between researchers: 12
Researcher 1(KS): excluded: 232, included: 15,
Researcher 2(SP): excluded: 226, included: 21,
After resolving conflicts:
Records excluded after title and abstract screening: 231
Reports assessed for eligibility in full text: 16
Reports excluded after full-text assessment: 2
Studies included in the systematic review: 14

Screening was performed independently by two reviewers. Disagreements were resolved by a third reviewer. Cohen's kappa was 0.641, indicating substantial agreement.
