# Supplementary material for: Micro- and Nanoplastics as Emerging Cardiovascular Risk Factors: A Systematic Review
Source: J Xenobiot. 2026 Jul 12;16(4):131. doi: 10.3390/jox16040131 (PMC13398113; doi:10.3390/jox16040131)
Supplement: Supplementary file 1 [file jox-16-00131-s001.zip › Supplementary File S7-Types and localization of microplastics in specimens, confounders, and reverse causation concern.pdf]

Supplementary File S7: Types and localisation of microplastics in specimens, confounders and reverse causation concern.

Authors	Specimen/Locatisation	All found plastics types	Dominant plastic types	Plastic concentrations	Detected biomarkers	Examined/associated diseases	Key (cardiovascular) findings	Source of microplastics/substances	Statistical model	Confounders adjustment	Important unmeasured confounders	Reverse causation concern
Yang et al. (July 2023) [19]	Pericardium, Epicardial and pericardial adipose tissue, myocardium, left atrial appendage	PET, PE, PU, PMMA, PA, PP, PVC, PC, PS	PET (77%), PU (12%) in	NA	NA	Cardiac surgery patients	MPs detected in examined tissues and blood; blood MPs profile changed after surgery	Operating room exposure/ surgical equipment	Descriptive analysis; comparison of MP types/counts before and after surgery	No multivariable adjustment	Age, sex, surgery type, underlying cardiac disease, comorbidities, medications, diet, SES, occupational exposure, iatrogenic/procedure-related exposure	Moderate/high - MPs measured during cardiac surgery; postoperative blood may reflect procedure-related exposure
	Blood		PET, PA	NA								
Massie et al. (September 2025) [20]	Atherosclerotic plaque of the common femoral artery	PE, PP, PS, ABS, SBR, PMMA, PC, PVC, PU, PET, N6, N66	NA	PE 2145 ± 3146,4 mg/g in sclerotic plaques<br>24,7 ± 11,4 mg/g in non-sclerotic	NA	Lower extremity peripheral arterial disease	~80× higher micro- and nanoplastics in femoral plaques vs healthy arteries	NA	Mann-Whitney U test; age-matched control comparison; correlations with age	Age matching only; no multivariable adjustment	Sex, smoking, diet, SES, occupational exposure, comorbidities, medications, iatrogenic exposure	High - MNPs measured in already diseased atherosclerotic plaques
Marfella et al. (March 2024) [7]	Arteriosclerotic plaque of the common carotid artery	PE, PVC	PE	NA	Elevated levels of IL-18, IL-1β, IL-6, TNF-α, CD3, CD68,<br><br>decreased collagen in plaques	Asymptomatic carotid artery stenosis	In patient with MNP-positive plaques: higher hazard of composite MI/stroke/all-cause death (HR 4.53)	NA	Cox regression for MACE; linear regression for plaque markers	Age, sex, BMI, cholesterol, HDL, LDL, triglycerides, creatinine, diabetes, hypertension, previous CVD	Smoking not in Cox model, diet, SES, occupational exposure, PM2.5/PM10, medications, iatrogenic exposure	High/moderate - MNPs measured in already existing atherosclerotic plaques

Liu et al. (May 2024) [21]	Arterial tissues, atherosclerotic plaques of the coronary arteries, carotid arteries, and the aorta	PET, PA-66, PVC, PE	PET	NA	NA	Coronary atherosclerosis / carotid atherosclerosis	Microplastics detected in all arterial samples; higher levels in plaque-containing coronary and carotid arteries	NA	ANOVA; Spearman correlations	No multivariable adjustment; age, BMI, BP, smoking, diet-related habits and air pollution were only explored in correlations	Sex, comorbidities, medications, SES, occupational exposure, iatrogenic exposure	High - MNPs measured in already diseased arterial tissues; plaque-containing arteries compared with aortic dissection tissue
Wu et al. (July 2023) [22]	Thrombi retrieved from the aorta and lower limb arteries	LDPE, Phthalocyanine, Hotasol Green GK, iron compound pigments	Iron based synthetic pigments	NA	Platelet count	Arterial dissection or acute arterial embolism of lower extremities	Microparticles detected in 16/26 thrombi; higher particle number associated with higher platelet levels	NA	Mann–Whitney/χ^2; multiple linear regression	Age, sex, smoking, drinking	Diet, SES, comorbidities, medications, occupational exposure, iatrogenic exposure	High - particles measured in thrombi after acute vascular events
Yang et al. (August 2024) [23]	Venous blood (in glass containers)	PE<br>PVC<br>PP<br>PP<br>PS<br>PA66	in control group: dominant PE (found in all samples, 74,40%)<br><br>in ACS group: dominant PE (found in 81/81 samples, 56,23%)	total MP 100,13 ug/g blood<br><br>total MP 161,65 ug/g blood	IL-6, IL-12p70<br>B-cell, NK-cell	Acute coronary syndrome	Higher blood MPs in ACS vs controls; higher in myocardial infarction vs unstable angina; higher MPs associated with higher SYNTAX score/vascular complexity	NA	Linear/Tobit regression; group comparisons	Age only in main regression analyses	Sex, smoking, diet, SES, occupational exposure, medications, detailed comorbidities, iatrogenic/procedure-related exposure	Moderate/high - cross-sectional; MPs measured after chest pain/ACS presentation

Wang et al. (April 2024) [24]	Thrombi retrieved from the cerebral arteries (IS), coronary arteries (MI) and deep veins of the lower limbs (DVT)	PA66<br>PVC<br>PE	for IS:PA66: 81,3%<br>PVC: 37,5%<br>PE: 25%<br><br>for MI<br>PA66: 40%<br>PVC: 20%<br>PE: 20%<br><br>for DVT:<br>PA66 100%<br>PVC 100%	mean total: 105,5 ug/g<br>mean PA66: 57,4μg/g<br>mean PVC: 59,6μg/g<br>mean PE: 77μg/g<br><br>mean total: 141,8 ug/g<br>mean PA66: 67,5μg/g<br>mean PVC: 14,4μg/g<br>mean PE: 134,3μg/g<br><br>mean PA66: 66,4 μg/g	D-Dimer	Ischaemic stroke / myocardial infarction / deep vein thrombosis	MPs detected in 80% of thrombi; higher MP levels linked to greater disease severity; D-dimer higher in MP-detected group	NA	Group comparisons; multiple linear regression	Age, sex	Smoking, diet, SES, occupational exposure, comorbidities, medications, iatrogenic/procedure-related exposure	High - MPs measured in already formed thrombi after IS/MI/DVT
Zhang et al. (August 2025) [30]	Umbilical cord blood (in glass containers)	PC<br>PE<br>PMMA<br>PP<br>PVC<br>PS	control<br>PE<br>PVC<br>PMMA	control<br>mean total: 82.44(41.36)<br>mean PE: 75.55mg/kg (40.67)<br>mean PVC: 5.509(3.288)<br>mean PMMA: 0.6857mg/kg (1.601)	NA	PIH	Higher total MPs, in umbilical cords of PIH cases	Questionnaire-assessed; associated with plastic bottles, plastic cutlery, and takeout meals	Group comparisons; logistic regression; correlation analyses	No comprehensive multivariable adjustment for MP-PIH association; lifestyle variables assessed, but none remained significant in multivariate model	Age, BMI, parity, GDM, diet, SES, occupational exposure, medications, antihypertensive drugs, infusion sets/iatrogenic exposure	Moderate - MPs measured at delivery after PIH status was already established

			PIH<br>PE<br>PVC<br>PC	PIH<br>mean PE:<br>115.1mg/kg (54.45)<br>mean PVC:<br>3.837(1.820)<br>mean PC:<br>0.9980mg/kg<br>(1.850)								
Wang et al. (January 2025) [25]	Fecal samples (only human group analyzed)	PA<br>PU<br>CPE<br>PVC<br>ACR<br>PP<br>FKM	PA (39.80%),<br>PU (14.98%),<br>CPE (14.94%),<br>PVC (4.18%),<br>ACR (4.16%),<br>PP (3.25%),<br>FKM (2.99%)	NA	NA	Hypertension	Higher fecal MPs in hypertension	NA	Group comparison; experimental mouse validation	Age matching; basic clinical variables compared	Sex, diet, SES, occupational exposure, medications, comorbidities, iatrogenic exposure	Moderate - human part cross-sectional; MPs measured after hypertension status was present
Zhang et al. (February 2025) [18]	Coronary artery blood and thrombi from coronary arteries	PVC<br>PE<br>PS<br>PA66<br>PP<br>PMMA<br>PA6<br>PC<br>PET<br>PLA<br>PBAT	PVC (95,4%)<br>PE (71,8%)<br>PA66 (61,8%)<br>PS (43,6%)	PE: 45,61 ± 26,25 mg/kg<br>PA66: 20,89 ± 12,87 mg/kg<br>PVC: 10,1 ± 7,7 mg/kg<br>PS: 2,47 ± 6,38 mg/kg	IL-1β, IL-6, IL-18, TNF-α	MACE in patients with MI	Higher coronary PVC linked to higher MACE risk, showed a dose-response relationship, and correlated with inflammatory activation	NA	Logistic regression for MACE; correlation analyses	alcohol, hypertension, diabetes	Diet, SES, occupational exposure, medication use, iatrogenic/procedure-related exposure, MNP sources	MNPs measured during/after MI; thrombi already formed
			PVC: (100%)<br>PA66: (85,7%)<br>PE: (76,2%)<br>PS: (47,6%)<br>PP: (4,8%)	PA66: 32,28 ± 14,57 mg/kg<br>PE: 18,33 ± 10,13 mg/kg<br>PVC: 15,67 ± 13,26 mg/kg<br>PS: 1,26 ± 0,67 mg/kg								

Yan et al. (December 2023) [26]	Fecal samples	PS<br>PE<br>Poliester:<br>PP<br>PA<br>PET<br>PAM	NA	for healthy patients:<br>mean total: 0,8 particles/g dw<br>PS: 0,3 particles/g dw<br>PP: 0,25 particles/g dw	NA	Vascular calcification	Higher fecal MPs, PP and PS in vascular calcification; calcification score positively correlated with total MPs, PP and PS	Questionnaire-assessed; bottled water, takeout food, dust exposure	Group comparisons; correlation analyses	No multivariable adjustment; age, sex and clinical variables compared only	Age, diet, SES, occupational/dust exposure, medications, comorbidities, smoking, iatrogenic exposure	Moderate - cross-sectional; MPs measured after VC status was already present
			PS: 42,4%<br>PE: 16,3%<br>Poliester: 15,7%<br>PE: 16,3%,.	for patients with VC (vascular calcification):<br>mean total:<br>PS: 1,4 particles/g dw<br>PP: 0,5 particles/g dw								
Cui et al. (August 2025) [27]	Arteriosclerotic plaques from the common carotid artery	PE<br>PP<br>PVC<br>SBR<br>PS<br>PMMA<br>PET<br>PA6, PA 66<br>PC<br>PU<br>ABS	PP (23,1%),<br>PE (20,3%),<br>SBR (19,8%)<br>PVC (18,5%)	Median MP: 432,9 µg/g	TG, LDL-C, HDL-C, ApoB	Carotid artery stenosis	Tissue-specific microplastic accumulation in blood and carotid plaque, with associations between microplastics and adverse lipid markers	NA	Spearman correlations; multivariate linear regression for lipid biomarkers	Age, sex, BMI, smoking, diabetes	Diet, SES, occupational exposure, medications, hypertension/stroke history, iatrogenic exposure	High - MPs measured in already existing carotid plaque
	venous blood		PE (46,5%)<br>PVC (16,8%),<br>SBR (16,3%)<br>PP (9,2%	Median MP: 75,2 µg/g								
Lee at al. (December 2024) [28]	Venous blood	PS<br>PP<br>PE<br>PET<br>PA	NA	NA	aPTT, CRP, fibrynogen	Blood coagulation and potential cardiovascular risks	Higher MPs load associated with increased aPTT, CRP, and fibrinogen	Questionnaire-assessed Lifestyle-related factors	Group comparisons; multivariate linear regression for coagulation/inflammatory markers	Age, sex, education, job, marital status, smoking, alcohol, physical inactivity	Diet, SES only partly, occupational exposure, medications, comorbidities, iatrogenic exposure	Low/moderate - cross-sectional; MPs and coagulation markers measured at the same time

Yu et al. (October 2024) [29]	Arterial blood	PVC<br>PA66<br>PS<br>PP	NA	for control group: mean total 79,82 ± 31,73 μg/g<br>PVC 60.80 ± 19.83 μg/g<br>PA 66: 19.03 ± 13.54<br>―――<br>for ECAS: mean total: 174,89 ± 24,95 μg/g<br>PVC: 134.79 ± 23.25 μg/g<br>PA 66 40.03 ± 7.31μg/g	D-dimer, thrombin time, homocysteine	Extracranial carotid artery stenosis (ECAS)	Higher blood microplastics in ECAS vs controls; MPs level positively correlated with stenosis severity	NA	Group comparisons; Pearson correlation; linear regression	Age, sex	Smoking, diet, SES, occupational exposure, medications, comorbidities, lifestyle plastic exposure, iatrogenic exposure	Moderate/high - cross-sectional; MPs measured after ECAS was already present

ABS acryloni-trile–butadiene–styrene; ACS - acute coronary syndrome; ANOVA - analysis of variance; ApoB - apolipoprotein B; aPTT - activated partial thromboplastin time; BMI - body mass index; BP - blood pressure; CRP - c-reactive protein; CVD - cardiovascular disease; DSA - digital subtraction angiography; DVT deep vein thrombosis; ECAS - extracranial carotid artery stenosis; FKM Fluor rubber; GDM - gestational diabetes mellitus; HDL - high-density lipoprotein cholesterol; HR - hazard ratio; IL- interleukin; IS ischaemic stroke; LDL - low-density lipoprotein cholesterol; LDPE Low-density polyethylene; MACE major adverse cardiovascular events; MI myocardial infarction; MNP microplastics and nanoplastics; MP(s) microplastic(s); N66 Poliamide 66; NA not assessed; ND - not demonstrated; PA6 polyamide 6; PA66 polyamide 66; PAD - peripheral artery disease; PBAT polybutylene adipate terephthalate; PBAT Polybutylene adipate terephthalate; PC polycarbonate; PE polyethylene; PET polyethylene terephthalate; PIH - pregnancy induced hypertension; PLA Polilactic acid; PLA polylactic acid; PM2.5, particulate matter with an aerodynamic diameter ≤ 2.5 μm; PM10, particulate matter with an aerodynamic diameter ≤ 10 μm; PMMA polymethyl methacrylate; PP polypropylene; PS polystyrene; PU Poliuretan; PVC polyvinyl chloride; SBR styrene-butadiene rubber; SES - socioeconomic status; TG - triglycerides; TNF-α - Tumor Necrosis Factor-α; VC - vascular calcification;
