# Supplementary material for: Micro- and Nanoplastics as Emerging Cardiovascular Risk Factors: A Systematic Review
Source: J Xenobiot. 2026 Jul 12;16(4):131. doi: 10.3390/jox16040131 (PMC13398113; doi:10.3390/jox16040131)
Supplement: Supplementary file 1 [file jox-16-00131-s001.zip › Supplementary File S8 - GRADE-informed certainty assessment.pdf]

Supplementary File S8: GRADE-informed certainty assessment

Evidence domain	Included studies	Evidence base	Main reasons for rating down	Certainty	Interpretation
MNPs in plaques and vascular or cardiac tissues	Yang et al., 2023 [19]; Massie et al., 2025 [20]; Marfella et al., 2024 [7]; Liu et al., 2024 [21]; Cui et al., 2025 [27]	Small observational tissue-based studies in selected clinical populations.	Risk of bias; indirectness; imprecision; incomplete confounder adjustment; reverse causation.	Very low	MNPs are detectable in cardiovascular tissues, but causality cannot be inferred.
MNPs in thrombi	Wu et al., 2023 [22]; Wang et al., 2024 [24]; Zhang et al., 2025 [18]	Small thrombus-based observational studies, measured after thrombus formation	Risk of bias; imprecision; indirectness; limited adjustment; strong reverse causation concern.	Very low	Particles may be present in thrombi, but their causal role in thrombosis is unclear.
Blood MNPs and cardiovascular disease or severity	Yang et al., 2023 [19]; Yang et al., 2024 [23]; Yu et al., 2024 [29]; Zhang et al., 2025 [18]; Cui et al., 2025 [27]; Lee et al., 2024 [28]	Cross-sectional, descriptive, observational blood-based studies.	Heterogeneous populations; limited temporal information; imprecision; inconsistent confounder adjustment.	Very low	Blood MNPs may be associated with disease status or biomarkers, but evidence is not causal.
Fecal MPs and vascular or cardiovascular outcomes	Yan et al., 2023 [26]; Wang et al., 2025 [25]	Small cross-sectional human fecal studies, partly supported by animal experiments.	Indirect matrix; very small samples; risk of bias; imprecision; limited adjustment; reverse causation.	Very low	Evidence is preliminary and hypothesis-generating.
MNPs and pregnancy-related	Zhang et al., 2025 [30]	Small observational maternal-fetal study.	Small sample; disease status already present; limited adjustment; indirectness; imprecision.	Very low	Evidence is insufficient to infer causality.

vascular outcomes					
MNPs and inflammatory, lipid, or coagulation biomarkers	Marfella et al., 2024 [7]; Cui et al., 2025 [27]; Lee et al., 2024 [28]; Zhang et al., 2025 [18]; Yan et al., 2023 [26]	Mostly surrogate biomarker analyses within small observational studies.	Surrogate outcomes; heterogeneity; imprecision; residual confounding; reverse causation.	Very low	Biomarker associations are exploratory and should not be interpreted as proof of mechanism.

GRADE interpretation key:

High certainty = the evidence provides strong confidence in the reported association.

Moderate certainty = the evidence provides moderate confidence, although the true association may differ from the reported estimate.

Low certainty = confidence in the evidence is limited, and the true association may be meaningfully different.

Very low certainty = confidence in the evidence is very limited, and the true association remains highly uncertain.
